# Exploring the Impact of Astaxanthin Supplementation in Conjunction with a 12-Week CrossFit Training Regimen on Selected Adipo-Myokines Levels in Obese Males

**DOI:** 10.3390/nu16172857

**Published:** 2024-08-26

**Authors:** Mohammad Ahmadi Moqaddam, Morteza Nemati, Marjan Mansouri Dara, Maha Hoteit, Zahra Sadek, Akbar Ramezani, Mahboubeh Khak Rand, Asieh Abbassi-Daloii, Zhaleh Pashaei, Abdullah Almaqhawi, Omid Razi, Kurt A. Escobar, Rashmi Supriya, Ayoub Saeidi, Hassane Zouhal

**Affiliations:** 1 Department of Physical Education and Sport Science, Science and Research Branch, Islamic Azad University, Tehran 1477893855, Iran; ahmadi.moqaddam@gmail.com (M.A.M.); mrj.md@yahoo.com (M.M.D.); 2 Department of Biomechanics and Sports Injuries, Faculty of Physical Education and Sports Sciences, Kharazmi University, Tehran 1571914911, Iran; mortezanemati24@yahoo.com; 3 Food Science Unit, National Council for Scientific Research of Lebanon (CNRS-L), Beirut 11-8281, Lebanon; m.hoteit@ul.edu.lb; 4 Section 1, Faculty of Public Health, Lebanese University, Beirut 6573, Lebanon; zahrasadek81@hotmail.com; 5 Laboratory of Motor System, Handicap and Rehabilitation (MOHAR), Faculty of Public Health, Lebanese University, Beirut 6573, Lebanon; 6 Ayatollah Amoli Branch, Department of Exercise Physiology, Islamic Azad University, Amol 6134937333, Iran; akbarramzani432@gmail.com (A.R.); mahboubeh.khakrand@yahoo.com (M.K.R.); abbasi.daloii@gmail.com (A.A.-D.); 7 Department of Exercise Physiology, Faculty of Physical Education and Sport Sciences, University of Tabriz, Tabriz 5166616471, Iran; pashaei.zh@gmail.com; 8 Department of Family Medicine and Community, College of Medicine, King Faisal University, Al Ahsa 31982, Saudi Arabia; aalmuqahwi@kfu.edu.sa; 9 Department of Exercise Physiology, Faculty of Physical Education and Sports Science, Razi University, Kermanshah 6714414971, Iran; omid.razi.physio@gmail.com; 10 Department of Kinesiology, California State University, Long Beach, CA 90840, USA; kurt.escobar@csulb.edu; 11 Centre for Health and Exercise Science Research, Hong Kong Baptist University, Kowloon Tong, Hong Kong 999077, China; 12 Academy of Wellness and Human Development, Faculty of Arts and Social Sciences, Hong Kong Baptist University, Kowloon Tong, Hong Kong 999077, China; 13 Department of Physical Education and Sport Sciences, Faculty of Humanities and Social Sciences, University of Kurdistan, Sanandaj 1517566177, Iran; 14 M2S (Laboratoire Mouvement, Sport, Santé)—EA 1274, Université Rennes, 35044 Rennes, France; hassane.zouhal@univ-rennes2.fr; 15 Institut International des Sciences du Sport (2I2S), 35850 Irodouer, France

**Keywords:** exercise, astaxanthin supplementation, myokines, obesity

## Abstract

Objective: Obesity is associated with an exacerbated metabolic condition that is mediated through impairing balance in the secretion of some adipo-myokines. Therefore, the objective of the present study was to explore the impact of astaxanthin supplementation in conjunction with a 12-week CrossFit training regimen on some selected adipo-myokines, insulin insensitivity, and serum lipid levels in obese males. Material and Methods: This study is a randomized control trial design; 60 obese males were randomly divided into four groups of 15, including the control group (CG), supplement group (SG), training group (TG), and combined training and supplement group (TSG). The participants were subjected to 12 weeks of astaxanthin (AST) supplementation [20 mg/d capsule, once/d] or CrossFit training or a combination of both interventions. The training regimen comprised 36 sessions of CrossFit, each lasting 60 min, conducted three times per week. The metabolic indices, body composition, anthropometrical, cardio-respiratory, and also some plasma adipo-myokine factors, including decorin (DCN), activin A, myostatin (MST), transforming growth factor (TGF)-β1, and follistatin (FST), were examined 12 and 72 h before the initiation of the main interventional protocols, and then 72 h after the final session of the training protocol. Results: There was no significant difference in the baseline data between the groups (*p* > 0.05). There were significant interactions between group x time for DCN (η^2^ = 0.82), activin A (η^2^ = 0.50), FST (η^2^ = 0.92), MST (η^2^ = 0.75), and TGFB-1 (η^2^ = 0.67) (*p* < 0.001 for all the variables). Significantly changes showed for DCN in TSG compared to TG and SG and also TG compared to SG (*p* = 0.0001); for activin A in SG compared to TG (*p* = 0.01) and TSG (*p* = 0.002); for FST in SG compared to TG and TSG (*p* = 0.0001), also in TSG compared to TG (*p* = 0.0001); for MST in SG, TG, and TSG compared to CG (*p* = 0.0001) and also in TSG compared to SG (*p* = 0.0001) and TG (*p* = 0.001); for TGFB-1 in SG, TG, and TSG compared to CG (*p* = 0.0001) and also TSG compared to SG (*p* = 0.0001) and TG (*p* = 0.001). Conclusions: The 12-week CrossFit training concurrent with AST supplementation reduced anthropometric and metabolic factors and also serum lipid levels while producing positive changes in body composition and cardiovascular factors. Increased FST and DCN and reduced activin A, MST, and TGF-β1 were other affirmative responses to both interventions.

## 1. Introduction

A positive energy balance, which stems from high energy consumption in proportion to energy expenditure, causes fat accumulation in adipose tissues, especially white adipose tissue, leading to obesity in the long term [1]. The adipocytes in obesity gradually undergo hypertrophy and hyperplasia followed by inflammation through attracting systemic monocytes/macrophages. Infiltrated macrophages in the white adipose tissue release some pro-inflammatory cytokines culminating in a phenomenon, namely, meta-inflammation or chronic low-grade inflammation [2,3]. This produced hyper-inflammation in obesity is associated with insulin resistance (IR) in both adipose and peripheral tissues [4]. As for white adipose tissue, IR is paralleled with releasing free fatty acids into the circulation taken up by peripheral tissues, such as the liver and skeletal muscles, to exacerbate the condition of IR [5]. Accrued body fat, and also its inflammatory consequences, alter body composition and aerobic capacity by decreasing skeletal mass (muscle atrophy) [6]. Increased adiposity, or rather, the rate of adipose tissue and/or its distribution, is also associated with secreting some signaling molecules including myokines and adipocytokines, termed adipo-myokines, from adipocytes and other metabolic tissues, which leads to metabolic syndromes such as IR, glucose intolerance, and hyperlipidemia [7]. 

The transforming growth factor (TGF)-β superfamily is composed of 33 members including TGF-β1-3, activins, myostatin (also known as growth differentiation factor-8 [GDF8]), and bone morphogenetic proteins (BMPs); among them, TGF-β1, activin A, and myostatin (MST) are the components with an intimate relationship with obesity [8]. Various mechanisms have been highlighted to increase their expression and also secretion into circulation related to obesity, including inflammation and accumulated lipids in adipose and muscle tissues [9,10,11]. It also should be mentioned that these factors have a facilitating effect on the secretion of each other [12,13,14]. The majority of obese and type 2 diabetic individuals and also ob/ob and db/db animals are faced with an imbalance/increase in the release and concentration of TGF-β members, probably as a compensatory mechanism [9,11,15,16]. Such released alterations associated with obesity in adipocytokines/myokines intensify metabolic conditions, such as IR, hyperglycemia, and hyperlipidemia, through influencing adipose, skeletal muscle, hepatic, and pancreatic tissues [17,18].

Increased energy expenditure is the most common intervention against obesity [19]. Elevated brown adipose tissues and also increased muscle mass partly belong to a procedure to escalate body energy expenditure [20]. As obesity-related lipid accumulation can change adipokine and myokine release, physical exercise along with some salutary alterations in adipose and skeletal muscle tissues is mediated by increasing some other adipokines/myokines [21]. Follestatin (FST) and decorin (DCN) are two local and secretory proteins whose concentrations change with positive energy balance [22,23]. FST is a glycoprotein enacting a conflicting role against TGF-β superfamily ligands and also its circulating concentration, which is released from the liver, and is dependent on increased energy demands during acute exercise and long-term fasting [23]. Elevated tissue and circulating levels of FST are accordant with brown adipose tissue [24] and increasing muscle mass [25]. Additionally, DCN is a small leucine-rich proteoglycan overexpressing in adipose tissue and in small amounts in skeletal muscles, and its expression and concentration in obese animals and patients is also increased [22]. Therefore, DCN may have a role in the normal function of adipose tissue and also in the pathogenesis of adiposity. It has been disclosed that physical exercise increases the production of FST and DCN levels, and thereby maintains muscle mass and mitigates fat accrual. The major mechanism of this resultant function is referred to as the inhibition of the signaling of three TGF-β members, including TGF-β1, MST, and activin A [26,27,28,29,30]. With respect to the above-mentioned, it is critical to adopt the mode of physical exercise with the characteristics of both preventing lipid accumulation and maintaining skeletal muscle mass, as it is a large metabolic organ playing essential roles in internal body homeostasis, energy expenditure, and insulin sensitivity [31].

CrossFit is categorized as high-intensity functional training (HIFT), which is defined by a mixed approach that integrates several aspects of fitness, such as endurance and strength [32]. This type of training includes workout sequences with rest periods in between sets [33]. This kind of training has acute physiological consequences that include elevated blood lactate levels [34,35], elevated testosterone, improved cortisol promotion [36], and elevated IL-6 and IL-10 activity [33]. It also triggers adaptive reactions that include higher muscular endurance, better aerobic capacity, increased lean body mass, better body fat utilization, or decreased body fat [32]. All of these changes associated with CrossFit training are beneficial for improving the detrimental effects related to obesity as well as increased lipid oxidation. The utilization of dietary supplementation concurrent with physical exercise may expedite reaching the aim of improving the metabolic conditions produced by obese patients.

Astaxanthin (AST), a compound of 3, 3′-dihydroxy-β, β-carotene-4, 4′-dione extracted from *Haematococcus pluvialis* algae, functions as an anti-obesity agent by enhancing energy expenditure, specifically by promoting lipid metabolism [37]. Although it has been well documented that AST can eliminate the detrimental consequences triggered by obesity [38], there is scant evidence about its effects on changes in adipocytokines and myokines including TGF-β1, activin A, MST, FST, and DCN, which have cardinal roles during obesity. By having in mind that circulating changes in these factors can accordingly alter other metabolic factors and also to the best of our knowledge, there is no document about the circulating changes in these adipo-myokines in relation to CrossFit training, we were prompted to find out the circulating changes in some adipo-myokines and metabolic factors following 12 weeks of CrossFit training along with AST supplementation. Considering the positive effects of CrossFit and AST supplementation on obesity and the lack of studies that examine the simultaneous effects of CrossFit training and AST supplementation on the mentioned adipo-myokines, we designed this study for the first time to answer this question: Do CrossFit training and AST supplementation each have an effect on adipo-myokines? Can CrossFit training and AST supplementation improve adipo-myokines at the same time?

## 2. Methods

The study is a double-blind, parallel-group randomized control trial designed to compare the independent and combined effects of astaxanthin and CrossFit Training. The subjects were recruited in the research through the publication of the call. People whose body mass index (BMI) was greater than 30 kg/m^2^ and who had not engaged in any regular physical activity for the previous six months were included in the study. Individuals who did not consume alcohol and had no past medical history of endocrine, metabolic, or cardiovascular disorders were eligible for inclusion. The participants with joint diseases or physical limitations were excluded, as were those taking prescription drugs or supplements that could affect the metabolism of muscle and adipose tissue. A physical examination conducted by an examiner was completed by all the participants during the initial visit. Additionally, the study procedures were elucidated at this time, and the participants were provided with a written consent form and the Physical Activity Readiness Questionnaire (PAR-Q).

The study was approved by the National Research and Ethics Committee (Ethics code: IR.IAU.DAMGHAN.REC.1401.035) and the Iranian Registry of Clinical Trials (IRCTID: IRCT20151228025732N76). The procedures were conducted in accordance with the most recent version of the Declaration of Helsinki.

## 3. Experimental Design

**Figure 1:**
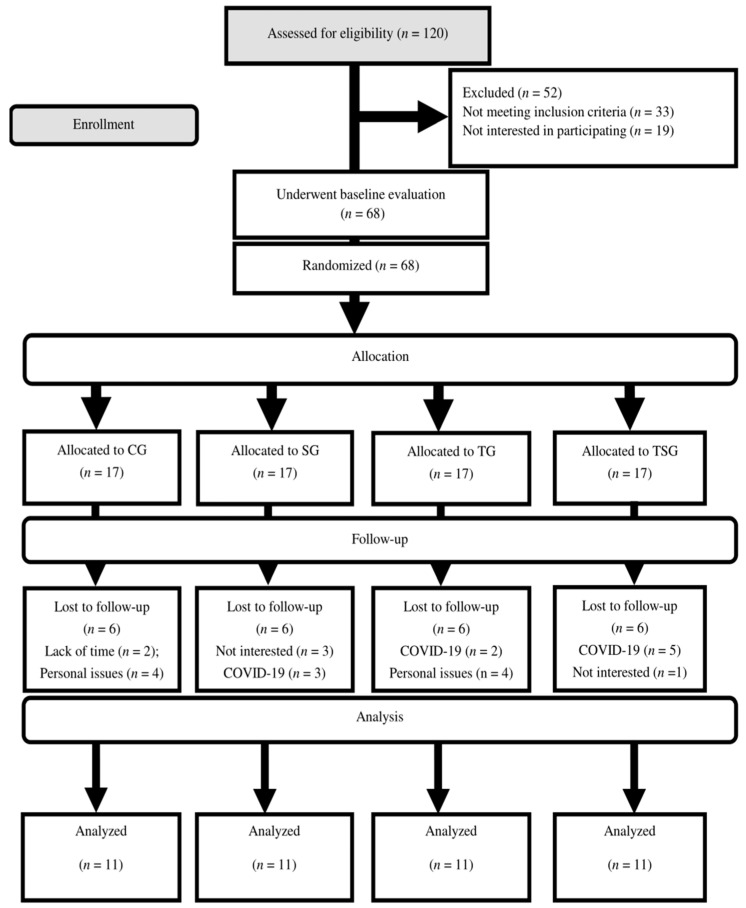
Flow chart of the participants.

A week before the training sessions started, the participants went through a familiarization session where all the study protocols were discussed. Each participant had their height, weight, and body composition measured before being randomized into one of four equal groups of 17 participants: training group (TG), control group (CG), supplement group (SG), and training + supplement group (TSG) (Figure 1). Randomization was performed according to a computer-generated allocation schedule through our research by using a fixed block size of eight (using a permuted block design with a computer random number generator). Astaxanthin and placebo were administered in a double-blind fashion in the guise of tablets that were identical in taste, color, and appearance. They were prepacked in bottles and placed into blurred study kits that were sequentially numbered for each subject according to the randomization schedule. The instructions for either the actual exercise program or the placebo exercise program were also placed into each kit. The allocation sequence was concealed from the research assistant enrolling and assessing the participants. Related kits were opened only after the enrolled participants completed all the baseline assessments and it was time to allocate the intervention. All the researchers and those involved in the concluded assessment were blinded to the group assignments and all the tests were then conducted by a research assistant blinded to the group assignments. Additionally, the training was supervised by two additional research assistants who were not involved in any other aspect of the study and were blinded to whether the subjects were on astaxanthin or placebo. All the data entry and statistical analysis were performed by another research assistant in a blinded manner.

There were 11 participants in each group at the end of the study after 24 volunteers from different groups withdrew for issues at work. Posttests were conducted for each group 48 h following the final session, and baseline evaluations were acquired 48 h prior to the start of the training protocols. An identical diet was followed by the participants in the training regimens 48 h prior to the baseline and final measures.

In the third session, the measurements of VO2peak and body composition variables were given along with instructions on how to perform the training regimens. The two training groups (TG and TSG) started the 12-week exercise training program with three sessions per week after the baseline measures. Throughout the trial, the control group was told to continue living as they now did. Every measurement was taken in the same light (within about an hour) and with the same ambient temperature (about 20 °C and around 55% humidity).

## 4. Training Protocols

In this study, the high-intensity functional training (HIFT) program was CrossFit, with a total of 36 sessions that lasted up to 60 min each. A CrossFit trainer with certification oversaw all the aspects of the training sessions. During the first two training sessions, the participants were introduced to basic activities that are frequently used in CrossFit training. These motions included pull-ups, kettlebell swings, medicine ball cleans, squats, deadlifts, presses, jerks, and barbell workouts. The first two days were spent without doing any extra exercise.

From day three onwards, every training session adhered to a set schedule that comprised five to thirty minutes of the Workout of the Day (WOD), which was performed at a high level of intensity based on each person’s capacity and fitness level, and ten to fifteen minutes of stretching and warm-up. The workout included bodyweight movements like squats and pull-ups, weightlifting activities like kettlebell swings and front squats, and aerobic workouts like running and jumping rope. Using the CrossFit training template [39] and offered in single, couplet, or triplet modalities, these workouts were continuously altered and performed for time, repetitions, or weight.

Every participant had their own weights and movements prescribed and documented [40]. The participants’ completion times, rounds and repetitions completed, weights utilized, and any necessary deviations from the prescribed workout were recorded depending on the structure of the WOD. For the entire training group, the average times for each WOD and the total average WOD time per week were determined.

## 5. Supplementation of Astaxanthin and Placebo

After meeting the eligibility requirements, the participants were randomized to receive either a daily dosage of 20 mg of astaxanthin capsules (Marine Product Tech. Inc., Seongnam, South Korea) or an identical placebo, which was administered once a day with breakfast for a period of 12 weeks. The placebo was in the form of a 20 mg/day raw corn starch capsule. The need of consuming 80% or more of the supplied supplements was set down in the intervention’s definition of adherence.

## 6. Nutrient Intake and Dietary Analysis

Three-day food records (two weekdays and one weekend day) were obtained before and after the study to assess changes in habitual dietary intake over time. Each food item was individually entered into Diet Analysis Plus version 10 (Cengage, Boston, MA, USA), and total energy consumption and the amount of energy derived from proteins, fats, and carbohydrates were determined (Table 1).

## 7. Blood Markers

Every testing process followed the established guidelines and was carried out between the hours of 8 and 10 in the morning. Blood samples were obtained from the right arm during a fasting state 12 h and 72 h prior to the first exercise session, and 72 h after the previous session. Following their transfer to EDTA-containing tubes, these blood samples were centrifuged for 10 min at 3000 rpm and then refrigerated at −70 °C.

- The plasma concentrations of TGF-β, decorin, myostatin, follistatin, and myostatin were measured with the R&D Systems (Boston Biochem, Boston, MA, USA) enzyme-linked immunosorbent assay (ELISA) kits.
- According to the R&D Systems guidelines, plasma activin A was quantified using ELISA (R&D Systems DAC00B) with intra- and inter-assay coefficients of variation less than 5%.

## 8. Statistical Analysis

The sample size was selected to detect a statistical difference between the study variables at a 95% confidence interval (CI) and a power value equal to or greater than 80%. Data analysis was carried out using the SPSS software (version 24).

- The threshold for establishing statistical significance was a *p*-value of less than 0.05.
- All the data were characterized using descriptive statistics, which are expressed as means ± standard deviation. The Shapiro/Wilk test was utilized to assess the normality of the data distribution.
- An ANOVA repeated measures test conducted in two ways was used to determine the group × time interaction.
- One-way ANOVA was used to evaluate the baseline data for each of the four groups, and Fisher LSD post hoc tests were used. Pairwise comparisons were used to ascertain mean differences in the cases where an ANOVA indicated a significant difference.
- Classified as trivial (<0.2), small (0.2–0.6), moderate (0.6–1.2), large (1.2–2.0), and very large (2.0–4.0). ESs were given as partial eta-squared.

## 9. Results

Fifty-two of the initial 120 volunteers for the study were ineligible to participate. Following a review, 68 individuals with a mean age of 27.6 ± 8.4, height of 167.8 ± 3.1 cm, weight of 94.7 ± 2.0 kg, and BMI of 33.6 ± 1.4 kg/m^2^ were selected at random and placed into four distinct groups of 17.

Examining the changes between and within the groups for energy, carbohydrates, fat, and protein did not show significant differences between the groups and within the groups (*p* > 0.05).

The comparison of baseline values for DCN (*p* = 0.91), activin A (*p* = 0.30), FST (*p* = 0.99), MST (*p* = 0.75), and TGFB-1 (*p* = 0.46) showed that there were no significant differences among the four groups.

**Figure 2:**
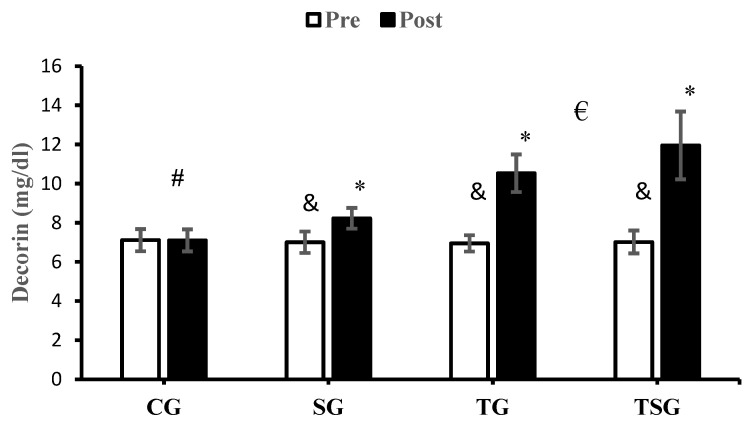
The mean ± standard deviation (SD) values of decorin before and after the training. * shows significant differences with the control group (*p* < 0.05). ^#^ depicts significant interaction between time and group (*p* < 0.05). ^€^ discloses significant interaction between time and group (*p* < 0.05). Control (CG), supplement (SG), training (TG), and training+ supplement (TSG) groups. ^&^ indicates significant differences with the pretest values (*p* < 0.05).

The changes in DCN levels in CG were not significant after 12 weeks (*p* = 0.97), while significant increases were observed in SG (*p* = 0.0001), TG (*p* = 0.0001), and TSG (*p* = 0.0001). The interactions of group and time for DCN (*p* = 0.0001, η^2^ = 0.82) were statistically significant. The results of the post hoc test revealed that after 12 weeks of training, the changes in DCN in SG (*p* = 0.017), TG (*p* = 0.0001), and TSG (*p* = 0.0001) were significantly different from the changes in CG. The increase in DCN in TSG was significantly higher in comparison to TG (*p* = 0.001) and SG (*p* = 0.0001). This significantly different increase was also reported in TG in comparison to SG (*p* = 0.0001) (Figure 2).

**Figure 3:**
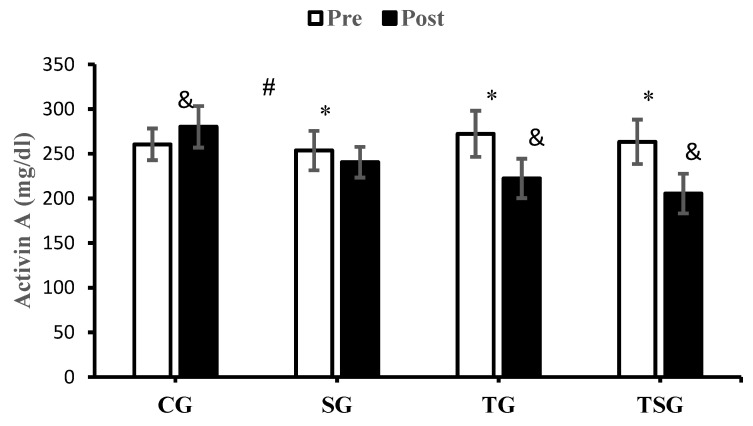
The mean ± standard deviation (SD) values of activin A before and after the training. * shows significant differences with the control group (*p* < 0.05). ^&^ indicates significant differences with the pretest values (*p* < 0.05). ^#^ discloses significant interaction between time and group (*p* < 0.05). Control (CG), supplement (SG), training (TG), and training+ supplement (TSG) groups.

There was a significant increase in activin A after 12 weeks of study in CG (*p* = 0.047), TG (*p* = 0.0001), and TSG (*p* = 0.0001) while the decrease in SG (*p* = 0.17) was not significant. The interactions of group and time for activin A (*p* = 0.0001, η^2^ = 0.50) were statistically significant. The results of the post hoc test revealed that after 12 weeks of training, the changes in activin A in SG (*p* = 0.021), TG (*p* = 0.0001), and TSG (*p* = 0.0001) were significantly different from the changes in CG. This significant difference was also reported in SG in comparison to TG (*p* = 0.01) and TSG (*p* = 0.002) while the decrease in activin A in TSG was not significantly higher in comparison to TG (*p* = 0.56) (Figure 3).

**Figure 4:**
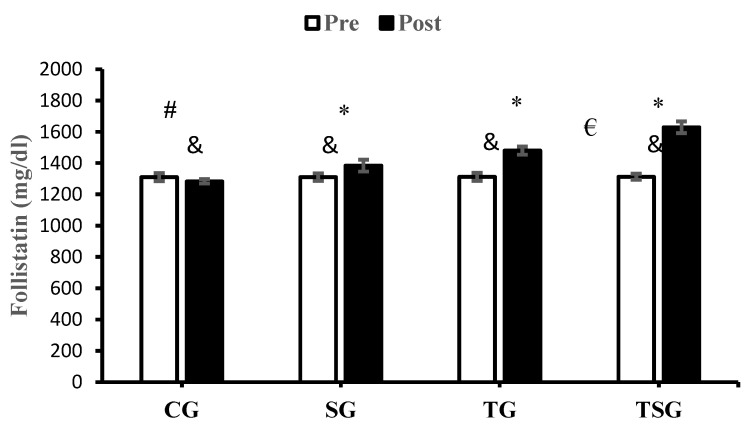
The mean ± standard deviation (SD) values for follistatin before and after the training. * indicates significant differences with the control group (*p* < 0.05). ^&^ shows significant differences with the pretest values (*p* < 0.05). ^#^ discloses significant interaction between time and group (*p* < 0.05). ^€^ depicts significant interaction between time and group (*p* < 0.05). Control (CG), supplement (SG), training (TG), and training+ supplement (TSG) groups.

After 12 weeks of study, FST decreased significantly in CG (*p* = 0.020) and increased significantly in SG (*p* = 0.0001), TG (*p* = 0.0001), and TSG (*p* = 0.0001). The interactions of group and time for FST (*p* = 0.0001, η^2^ = 0.92) were statistically significant. The results of the post hoc test revealed that after 12 weeks of training, the changes in FST in SG (*p* = 0.0001), TG (*p* = 0.0001), and TSG (*p* = 0.0001) were significantly different from the changes in CG. This significant difference was also reported in SG in comparison to TG (*p* = 0.0001) and TSG (*p* = 0.0001); also, the decrease in FST in TSG was significantly higher in comparison to TG (*p* = 0.0001) (Figure 4).

**Figure 5:**
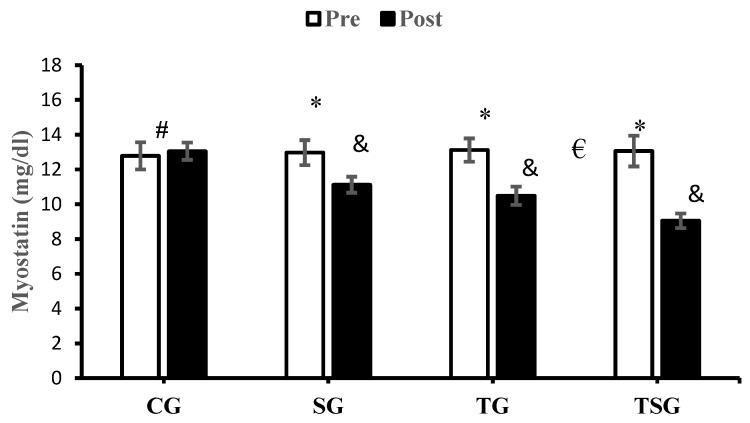
The mean ± standard deviation (SD) values for myostatin before and after the training. * indicates significant differences with the control group (*p* < 0.05). ^&^ significant differences with the pretest values (*p* < 0.05). ^#^ shows significant interaction between time and group (*p* < 0.05). ^€^ discloses significant interaction between time and group (*p* < 0.05). Control (CG), supplement (SG), training (TG), and training+ supplement (TSG) groups.

The results of the analyses showed that after 12 weeks of the study period, the increased levels of MST in CG were not significant (*p* = 0.34) while MST has been decreased significantly in SG (*p* = 0.0001), TG (*p* = 0.0001), and TSG (*p* = 0.0001). The interactions of group and time for MST (*p* = 0.0001, η^2^ = 0.75) were statistically significant. The pairwise comparison of the changes between the study groups showed that the changes in MST in SG (*p* = 0.0001), TG (*p* = 0.0001), and TSG (*p* = 0.0001) were significantly different from the changes in CG. This significant difference was also reported in TSG in comparison to SG (*p* = 0.0001) and TG (*p* = 0.001), but the decrease in MST in TG was not significantly higher in comparison to SG (*p* = 0.054) (Figure 5).

**Figure 6:**
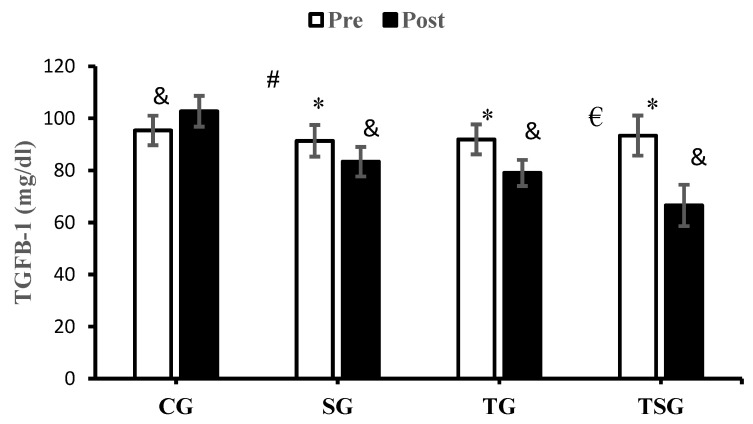
The mean ± standard deviation (SD) values for TGFB-1 before and after the training. * indicates significant differences with the control group (*p* < 0.05). ^&^ shows significant differences with the pretest values (*p* < 0.05). ^#^ discloses significant interaction between time and group (*p* < 0.05). ^€^ depicts significant interaction between time and group (*p* < 0.05). Control (CG), supplement (SG), training (TG), and training+ supplement (TSG) groups.

The posttest values of TGFB-1 were significantly different from the pretest in CG (*p* = 0.009), SG (*p* = 0.005), TG (*p* = 0.0001), and TSG (*p* = 0.0001). The interactions of group and time for TGFB-1 were significant (*p* = 0.0001, η^2^ = 0.67). The pairwise comparison of the changes between the study groups showed that the changes in TGFB-1 in SG (*p* = 0.0001), TG (*p* = 0.0001), and TSG (*p* = 0.0001) were significantly different from the changes in CG. This significant difference was also reported in TSG in comparison to SG (*p* = 0.0001) and TG (*p* = 0.001), but the decrease in TGFB-1 in TG was not significantly higher in comparison to SG (*p* = 0.20) (Figure 6).

## 10. Discussion

In the current study, we attempted to provide a transparent view of the changes in some adipo-myokine indices (DCN, activin A, FST, MST, and TGFB1) following a 12-week CrossFit training and AST supplementation in obese individuals. The results indicated that 12 weeks of CrossFit training alone and along with AST supplementation and even AST administration by itself could change the above-mentioned factors and these changes were significant between the interventional groups and control group.

Obesity is associated with increased circulatory metabolic factors, including hyperlipedemia, hyperinsuliemia, and hyperglycemia; it has been proposed that regular exercise training and taking food supplements are ideal strategies against obesity and related comorbidities.

It has been indicated that most of the negative metabolic effects of obesity are caused by the imbalance of adipo-myokines. Obesity is associated with increasing some members of the TGF-β family such as MST and activin A [8,41] and DCN [42] and diminished FST concentration [43]. Decorin is a small leucine-rich proteoglycan that increases in concentration in response to fat accumulation and its related inflammatory and metabolic changes during obesity [42]. Such local upregulation and increases in circulatory concentration in obese patients can regulate some functions, including the regulation of angiogenesis and vascular skeleton in adipose tissue [44]; the nullification/sequestration of 1q complement component through binding to it and protecting against its effects on IR [45]; playing as a decoy receptor for secretory adipocyte, namely resistin, that through this action reduces IR [46]; interaction with upregulated TGF-β during obesity to reduce its inflammatory and fibrotic actions; and the inhibition of adipogenic differentiation in adipose tissue [47]. In agreement with our results, it has been shown that DCN circulation and its muscular expression are elevated following both acute resistance exercise and chronic endurance training [26,29]. Its expression mediated by exercise is influenced by the intensity used in exercise, such that high-intensity exercise reduces its expression while moderate exercise upregulates DCN expression in skeletal muscle [48,49]. It has been reported that DCN expression is under the control of the balance of the tissue inhibitor of metalloproteinases-2 (TIMP-2) and matrix metalloproteinase-2 (MMP-2) in such a manner that a higher expression of MMPs is associated with more degeneration of DCN protein [50]. It has been revealed that this balance is deranged during high-intensity exercise in favor of a higher MMP-2 concentration [49]. Promoted DCN expression and its circulating levels during exercise may partly refer to the regulation by growth hormone (GH) since it has been disclosed that GH released in response to exercise was associated with increased serum DCN [51,52]. Some other documents reported that increased GH effects on DCN are mediated by simultaneous elevation in insulin-like growth hormone-1 (IGF-1) [51]. An increased DCN level following exercise has a beneficial effect on obesity-produced metabolic disorders through a multiplicity of mechanisms involving skeletal muscle. One of these mechanisms is precluding other adipo-myokines such as MST and TGF-β as an inhibitory factor of myogenesis, and also downregulating muscle-specific RING finger protein 1 (muRF1) and antrogin 1 as the degenerating components of muscle protein [27,53]. Elevating the upregulation of FST may be a pathway through which DCN can mitigate the above-mentioned factors [54]. With these functions, DCN upregulates myogenic MyoD protein to promote myoblast differentiation and as a result, muscle hypertrophy [55]. It has also been shown that AST increases MMPs, especially MMP-2, which thereby degenerates fibrotic components formed by TGF-β and inflammation conditions [56]. In this context, DCN is a component of the extracellular matrix (ECM) [57] that is released into the circulation by the action of MMPs. In this context, we found in the current study that DCN concentration is increased following 12 weeks of supplementation with AST. Thus, we can conclude that some positive effects of AST on muscle [58], which are associated with metabolic benefits, may rebound to increasing circulating DCN concentration. However, this part of the study needs to be further investigated in the future to help the obese community.

The attenuation of three members of the TGF-β family by blocking their receptors and downstream signals and also reducing their expression/concentration can protect the body against metabolic disorders [59]. Lifestyle changes, or rather, the contribution of regular exercise and sound nutrition can be a beneficial strategy to improve metabolic status [60]. Activin A is an inflammatory mediator and can change its expression and circulation following exercise [61], although there is a discrepancy in findings in this context (some showed an increase and some a decrease) [62]. However, it has been indicated that exercises with different intensities could mitigate the activin A concentration [62]. Although there is a lack of information about the response of activin A to exercise, limited previous reports pinpointed an increased expression of activin A secondary to exercise [61]. Conflicting with the previous findings, our finding showed a reduction in plasma activin A concentration following the 12-week CrossFit training. The probable mechanisms for such attenuation in the activin A concentration are referred to as the stress induced by exercise. The stress associated with exercise increases glucocorticoid secretion, which in turn mitigates activin A production [63]. On the other hand, endurance exercise reduces plasma glucose levels and negative energy balance. The established energy poverty negatively regulates activin A concentration [64]. Besides, resistance training also reduces the concentration of this TGF-β member by subjecting the exercising muscle to mechanical strain [65]. While increased activin A signaling is associated with the elevation of collagen III expression, the production of pro-inflammatory cytokines in macrophages can produce adipose fibrosis [66]. Reduced lipolysis is another upshot of the upregulation of activin A expression. Mitigating the expression of catecholamine receptors on adipose tissue by limiting the CCAAT enhancer binding protein alpha (C/EBPα) expression, increasing FFA accumulation in peripheral organs [67], mitochondrial dysfunction [68], and promoting muscle atrophy [69] may be some mechanisms whereby activin A attenuates lipolysis. In addition, modulating insulin secretion by pancreatic β-cells and suppressing the expression of transmembrane glucose transporter 4 (GLUT4) are other mechanisms to engender IR following increased activin A signaling [70].

MST, as a member of the TGF-β family, is a negative regulator of muscle mass, which is a leading tissue in promoting insulin-stimulated glucose. It should be mentioned that MST elevates muscle atrophy via protein degeneration induced by the increased activation of ubiquitin-proteases [71]. Compromising muscle glucose uptake through mitigating GLUT4 and AMPK is another mechanism that MST can use to worsen metabolic disorders related to obesity [72]. Last but not least, MST suppresses the differentiation of brown adipose tissues by inhibiting irisin expression in adipocytes [73]. The results related to the alteration of MST expression and its circulating concentration in response to resistance/strength and endurance training are conflicted in such a way that some reports pinpointed an increase [74] and others reported a decrease [75,76,77]. By the same token and in total agreement with our finding, the majority of the previous reports disclosed that both chronic and acute resistance and endurance exercises mitigate MST mRNA expression in skeletal muscle and its plasma levels [77,78,79,80]. Additionally, six-month moderate-intensity aerobic training [77] and eight-week resistance training [75] dampened the muscle and circulating levels of MST. Besides, it has been illustrated that its concentration abated in diabetic rats following four-week swim training [81]. Also, it has also been claimed that there is a positive relationship between muscle mass and MST concentration, suggesting skeletal muscle is the main source of MST release [82]. Accordingly, 12-month combined training in patients with kidney disease elevated plasma MST levels [83]. Increased MST induced by exercise has been ascribed to a normal response to promoted muscle mass through which this factor can be enacted as an inhibitory agent to excess muscle growth [84]. Such disparity between our finding and the previous investigations that reported an increase in this factor may reflect the used training modality, the time at which specimens were obtained, and the rate of incurred inflammation in the body during the exercise session. It has been revealed that training that uses more type II fibers type than type I fibers may be associated with a higher MST concentration [85]. Interestingly, it should be mentioned that if the blood samples are obtained instantly and up to 24 h after the exercise session, the MST levels are still high related to its baseline [78,79]. Besides, pro-inflammatory cytokines upregulate the expression of the MST gene [86]. The likely mechanisms whereby exercise reduces the expression and consequently, the concentration of MST levels may involve the following items: There has been a negative correlation between the concentration of irisin and FST with MST [87,88]. In this regard, 12-week combined training revealed that the serum concentration of both irisin and FST increased, while the serum MST level reduced [87]. Our results also were consistent with previous reports [89,90]. Increased levels of circulating irisin can activate the AKT signaling pathway, which consequently impacts MST synthesis [91]. Also, exercise training is associated with the increased secretion of IGF-1 and testosterone hormones. Both hormones activate the PI3K/PTEN/AKT pathway, which in turn mitigates circulating MST levels [92,93]. Testosterone additionally activates the Notch signaling pathway to elevate Mighty expression and as a result, inhibits the MST pathway in skeletal muscle [94]. The above-mentioned mechanisms inhibiting MST expression and its signaling can be accompanied by escalating muscle mass, promoting lipid oxidation, altering body composition, and improving glucose tolerance and insulin sensitivity.

Increased TGF-β1 in the aftermath of adiposity is accompanied by the activating of its downstream, known as Smad3. Smad3 can bind to both the gene promoters of PGC-1α and insulin in adipose tissue and pancreas, respectively. Upon binding, their transcription is blocked, which in turn reduces brown adipose tissue and insulin release from pancreatic cells [95,96,97]. In more general terms, the majority of the findings indicated an increased TGF-β1 expression in skeletal muscle following both endurance training and mechanical loading [98,99]. In contrary to our results, it has been shown that three different contractions, isometric, concentric, and eccentric, with four days of sciatic nerve stimulation altered the TGF-β1 mRNA levels of gastrocnemius muscle [100]. The promoted circulating levels of TGF-β1 following exercise have been ascribed to increased pro-inflammatory cytokines [101], consequent damages in skeletal muscle [102], compromised oxidative stress condition, and the reduced activity of antioxidant enzymes [103]. In any case, its overproduction is associated with forming fibrosis and muscle atrophy and also dampens metabolic adaptation to exercise [104,105]. In contrast to some previous studies, our finding showed a reduction in TGF-β1circulation after 12 weeks of CrossFit training that is concordant with previous evidence [99]. Exercise training reduces TGF-β1 expression through several mechanisms, including (1) mitigating oxidative stress and increasing antioxidant capacity which are responsible for ROS removal. This deed defies the function of ROS on the proteodegeneration of TGF-β1 from the latency-associated peptide/latent TGF-β1 binding protein (LAP/LTBP) complex [106,107]. It has been indicated that the mechanical loading of training protocol is accompanied by increasing Notch activation, which in turn blocks TGF-β1 signaling [108]. In addition, exercise training can inhibit the activation of NF-κB in macrophages, thereby attenuating TGF-β1 expression by macrophages [109].

The reduced activation of macrophages and their inflammatory mediators are accompanied by precluding the release of these members of the TGF-β family. The other mechanisms through which AST can suppress the expression of these family include increased antioxidant capacity [110], diminished ROS and oxidative stress [111], the dampened expression and activation of MMPs [112], the inhibited infiltration of inflammatory M1 macrophages into the tissues [113], and elevated circulating irisin [114].

FST is a monomeric glycosylated protein expressed in copious tissues including the testis, pituitary, muscle, liver, and adipose tissues [115]. In humans, circulating FST is often dependent on its release from the liver, although skeletal muscles and adipocytes have a critical role in this regard [30]. It has been suggested that its circulation is reduced in obese and diabetic patients [116]. Glucagon to insulin ratio is the main factor controlling FST secretion from the liver in such a manner that increased insulin concentration and its signaling into the liver reduces the expression and consequently, FST secretion through inactivating forkhead box protein O1 (FOXO1) [117]. Reduced expression in the white adipose tissue of obese individuals may be a compensatory response since FST increases adipogenesis through suppressing the inhibitory role of TGF-β members on adipogenesis [43]. Importantly, the increased concentration of TGF-β members like activin A and MST, as a compensatory pathway, reduces FST concentration, and thereby, the inhibitory effect of FST on the expression/concentration of TGF-β members may be eliminated [118]. However, FST is a stress-responding protein that responds to stressful conditions such as energy deficiency by increasing its expression and secretion [119]. It has been revealed that circulating FST levels are elevated following exercise sessions [30]. Exercise causes a negative energy balance which is associated with an increase in glucagon secretion and as a result, a promotion in FST release from the liver [120]. Furthermore, elevated glucagon devastates the complex of glucokinase regulator/glucokinase (GCKR-GCK) in the liver that in turn increases FST secretion [121,122]. Increased inflammation during exercise sessions may be another mechanism through which FST can be elevated in circulation [123]. Positive response to glucagon to insulin ratio, pro-inflammatory cytokines, and TGF-β members suggest the several roles of FST in relation to energy metabolism, immune regulation, and the protection of obese patients against the negative effects of TGF-β members. FST has enormous functions in the body and we can list some of them as follows: reduces serum levels of glucose, insulin, TGs, cholesterol, and FFAs; increases muscle mass; and also mitigates macrophage infiltration into the tissues and consequently, their pro-inflammatory production [118,124]. The above-mentioned effects induced by increased FST concentration can be accompanied by an improvement in metabolic disorders related to obesity.

In spite of the metabolic benefits resulting from the administration of AST, there is no document related to changes in FST concentration as a leading factor in providing metabolic improvement during or following AST supplementation that should be considered in future investigations. In any case, in the current study, we found an increase in FST after AST administration. This elevation is probably rebounded to reduced insulin concentration, as a main agent to control FST concentration, following AST supplementation [125] which attenuated the circulating members of the TGF-β family including activin A, MST, and TGF-β1, since these mitigating effects can release FST bound to these factors [56].

These circulating adipo-myokines were selected in this study because changes in each of them with any intervention influence many metabolic organs such as muscles, the liver, and the pancreas. The examination of the morphological and functional factors related to the metabolic disorders induced by obesity is another strength of the current study.

Our study had some limitations, including the following: First, our study does not include any female participants. Since females naturally have more adipose tissues and also less androgens influencing the changes in those adipo-myokines, it would be better to evaluate the differences in the changes in the metabolic and adipo-myokines factors between females and males. Second, we did not delve into the mRNA expression of adipo-myokines in at least adipose and muscle tissues. Third, the status of the immune system was not assessed, since this system is a main component to alter the expression and concentration of these adipo-myokines.

## 11. Conclusions

The 12-week CrossFit training along with AST supplementation improved metabolic factors related to insulin, glucose, and lipid profiles. Additionally, this 12-week exercise training supplemented with AST resulted in divergent findings in adipo-myokiness such that the plasma levels of DNC and FST were elevated while three members of the TGF-β family including activin A, MST, and TGF-β1 were diminished.

## Figures and Tables

**Table 1:** Mean (±SD) values of nutritional intake in the four study groups.

	CG			SG		TG	TSG	
	Pre	Post	Pre	Post	Pre	Post	Pre	Post
Energy (kcal/d)	2260 ± 47	2269 ± 56	2278 ± 101	2149 ± 100	2269 ± 117	2141 ± 117	2273 ± 157	2129 ± 126
CHO (g/d)	281 ± 31.4	283 ± 33.3	279.4 ± 27.1	261 ± 27.5	289 ± 48.6	261 ± 39.2	288 ± 38.6	259 ± 29.1
Fat (g/d)	82.2 ± 11.0	81 ± 9.8	86.5 ± 10.7	75 ± 11.2	83.4 ± 12.4	73.1 ± 11.2	80.8 ± 13.87	70.2 ± 11.3
Protein (g/d)	104 ± 12.0	106 ± 11.3	101 ± 13.5	93 ± 12.6	103 ± 14.8	94 ± 11.7	102 ± 14.5	90 ± 13.5

CG: control group; SG: supplement group; TG: training group; TSG: training supplement group.

## Data Availability

The data presented in this study are available within the manuscript.
